# Feasibility of dpFAMM flap in tongue reconstruction after facial vessel ligation and radiotherapy—case presentation

**DOI:** 10.1186/s12957-022-02554-w

**Published:** 2022-03-12

**Authors:** Michał Gontarz, Jakub Bargiel, Krzysztof Gąsiorowski, Tomasz Marecik, Paweł Szczurowski, Jan Zapała, Grażyna Wyszyńska-Pawelec

**Affiliations:** Department of Cranio-Maxillofacial Surgery, Jagiellonian University Medical College, University Hospital, Jakubowskiego 2 Street, 30-688 Cracow, Poland

**Keywords:** FAMM flap, Facial artery musculomucosal flap, Bozola flap, Tongue cancer, Reconstruction, Salvage surgery, Buccinator myomucosal flap, Tongue reconstruction, Tongue squamous cell carcinoma, dpFAMM flap

## Abstract

**Background:**

Salvage surgery with reconstruction of the second and next primary tongue cancer remains difficult, especially after earlier neck dissection and radiotherapy. In the current report, we describe the feasibility of the extended, double-pedicled facial artery musculomucosal (dpFAMM) flap in the reconstruction of the patient with second primary tongue squamous cell carcinoma, after facial vessel ligation and radiotherapy.

**Case presentation:**

An 81-year-old female patient was operated on due to tongue squamous cell carcinoma (SCC) on the left side T3N1M0 in 2019. Bilateral selective neck dissection with tongue reconstruction was performed by island FAMM flap. The patient also suffered from synchronous mucinous breast carcinoma treated with tamoxifen. The second primary SCC of the tongue on the opposite (right) side was detected in 2020. The patient did not agree to surgical treatment; therefore, radiotherapy was performed. The local recurrence of the tongue cancer of the right side was treated surgically in 2021. Salvage surgery comprised hemiglossectomy and dpFAMM flap reconstruction with uneventful postoperative follow-up.

**Conclusions:**

This case presentation proved that dpFAMM flap can be used in salvage surgery and reconstruction even in patients after ligation of facial vessels, irradiation, and in the course of hormone therapy. The flap is easy to handle, has good vascularity, and comprises a predictable method of reconstruction, especially for patients with severe comorbidities.

## Background

The incidence of synchronous and metachronous second oral cancer is increasing due to a longer lifespan and improvement of oncological therapy [1]. However, surgical treatment and reconstruction of second and next primary cancer in the oral cavity remain difficult, especially after previous neck dissection and radiotherapy. Defects of the tongue might be reconstructed by local flaps, such as the facial artery musculomucosal (FAMM) flap and its modifications [2–7]. The FAMM flap is an axial flap based on the facial artery and is useless in case of facial vessel ligation. In such cases, the reconstruction should be converted to a Bozola flap [8]. Extended, double-pedicled FAMM (dpFAMM) flap owns modification of FAMM flap with facial and buccal vessel blood supply [3].

In the current report, we describe the feasibility of the dpFAMM flap in the reconstruction in the patient with second primary tongue squamous cell carcinoma, after facial vessel ligation and radiotherapy.

## Case presentation

**Fig. 1:**
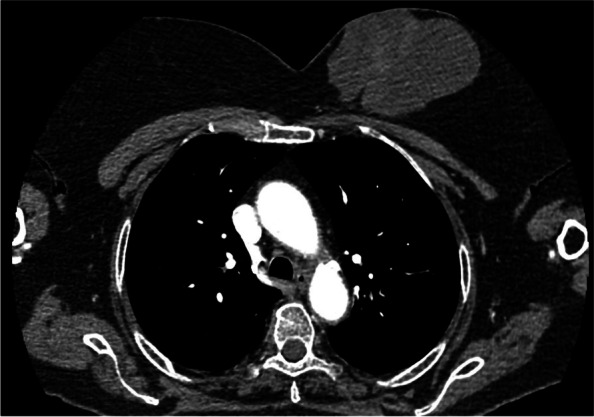
CT axial view of the chest showing huge breast cancer on the left side

**Fig. 2:**
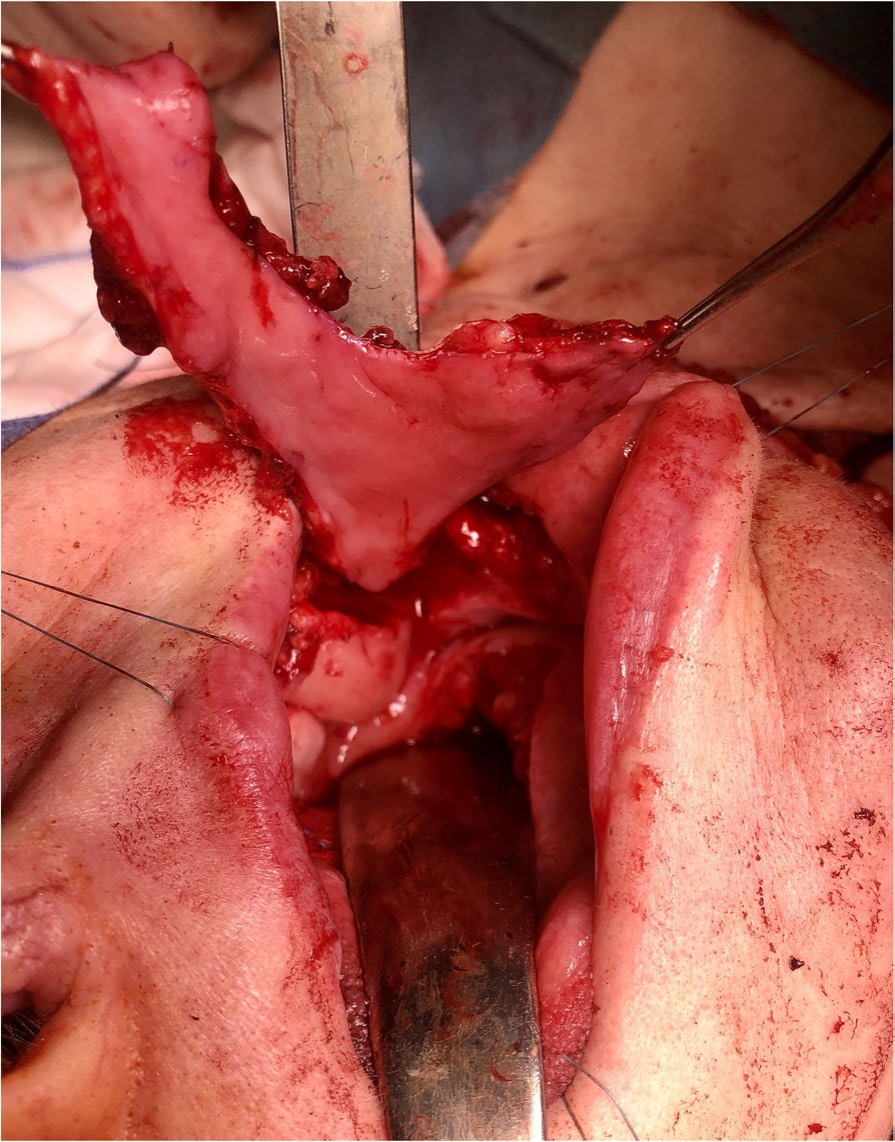
The harvested trilobed iFAMM flap

**Fig. 3:**
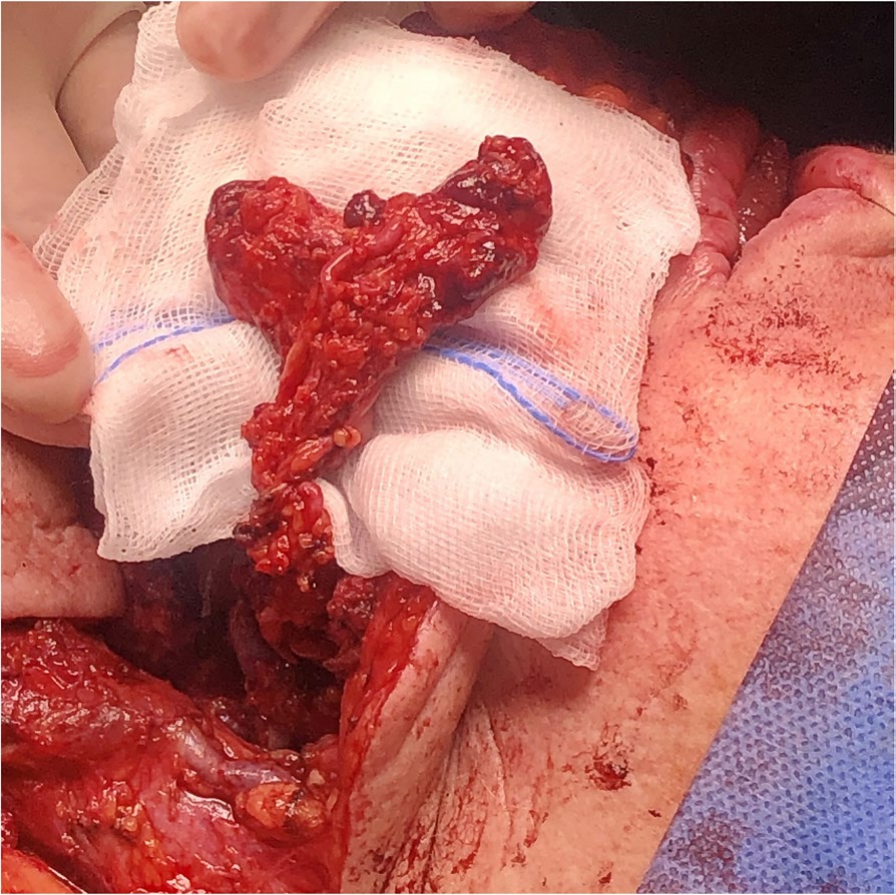
Transposition of the iFAMM flap with the facial vessel pedicle over the mandible

**Fig. 4:**
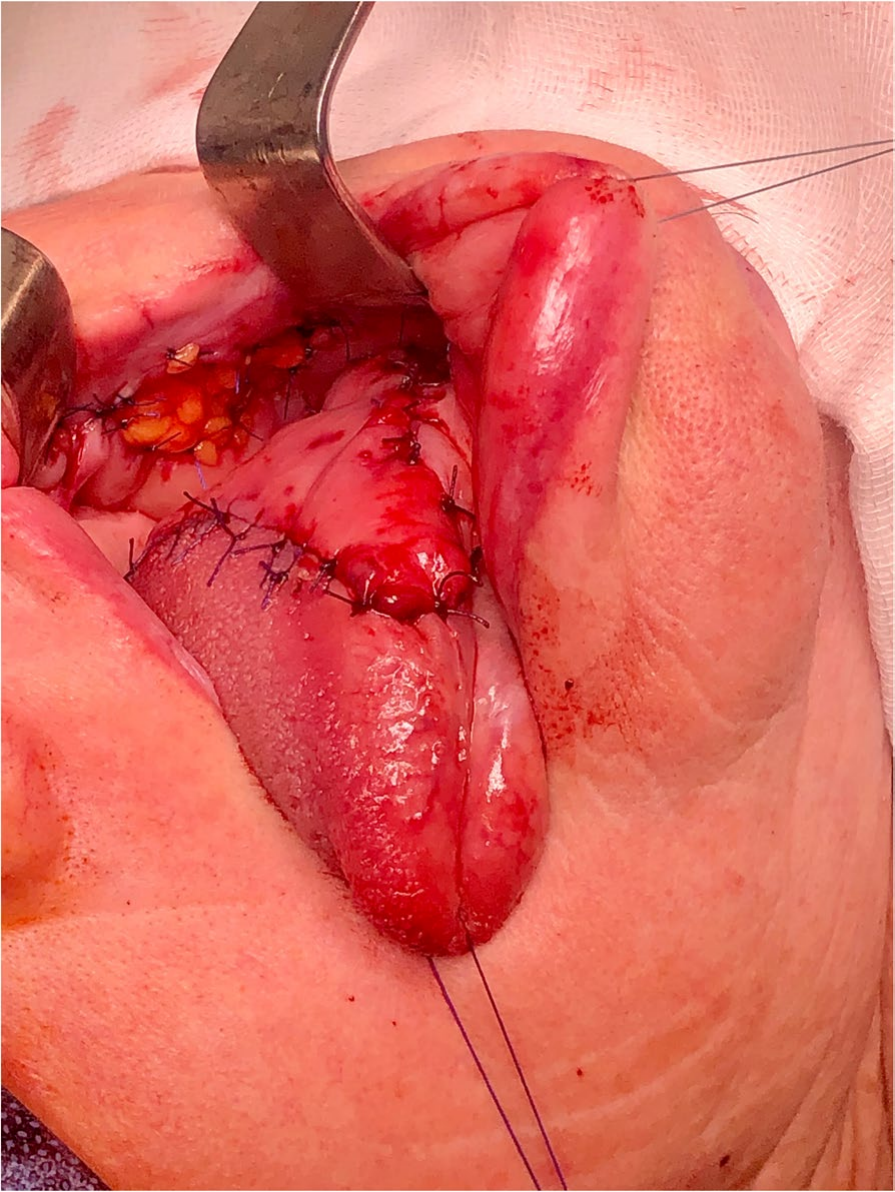
Immediate postoperative result of tongue reconstruction with iFAMM flap

In November 2019, an 81-year-old female patient was admitted to the Department of Cranio-Maxillofacial Surgery of the Jagiellonian University in Cracow due to tongue squamous cell carcinoma (SCC) on the left side cT3N1M0. Clinical examination and computed tomography (CT) revealed a synchronous left breast tumor (Fig. 1). Biopsy from the breast tumor revealed mucinous carcinoma, and diagnostic imaging excluded dissemination. After tumor board consultation, we decided to start the treatment with surgical excision of tongue SCC and bilateral neck dissection. Selective bilateral neck dissection (level I–IV ipsilateral and I–III contralateral) was performed by a horizontal neck fold incision. During neck dissection, the facial vessels on the left side were preserved for an island FAMM (iFAMM) flap. However, the facial vessels on the right side were ligated. Tongue cancer was excised with margins control by frozen section examination. Tongue reconstruction with an iFAMM flap was performed according to the technique described by Joseph et al. (Figs. 2, 3, and 4) [4]. The healing process was uneventful. Histopathological examination revealed squamous cell carcinoma G1 resected with inadequate distal margin (2mm) and metastasis in one lymph node (IIa cervical level) on the left side (pT3N1). The patient was qualified for postoperative radiotherapy and radical mastectomy with axillary node dissection. However, the patient did not give her consent for the proposed treatment.

**Fig. 5:**
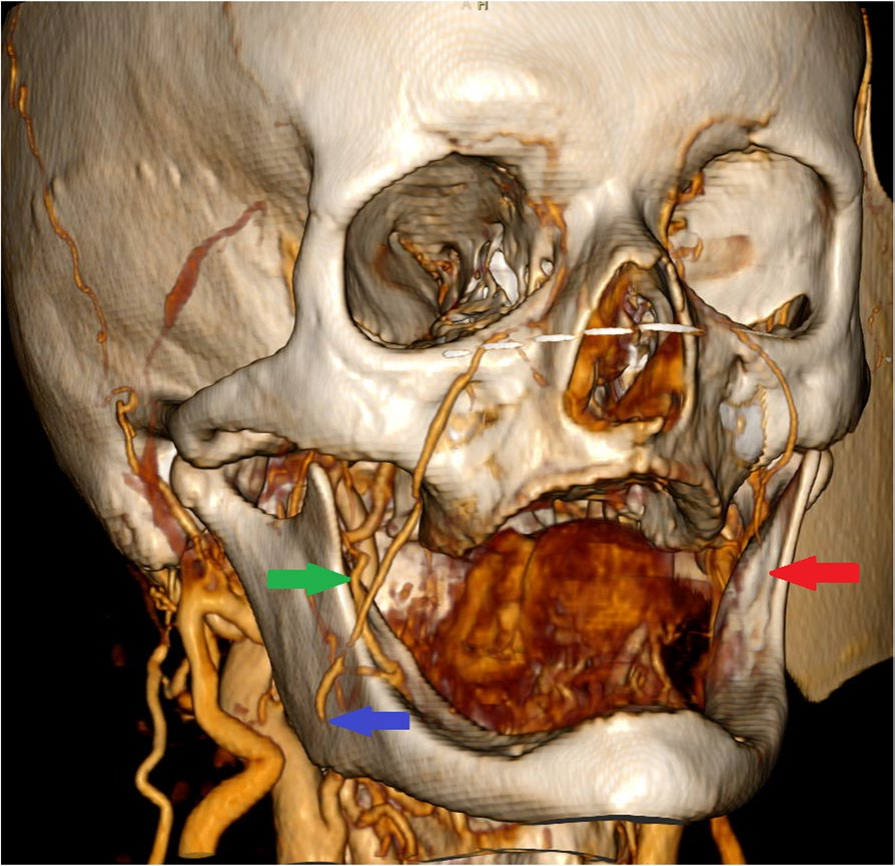
CT with 3D reconstruction showing stump of ligated facial artery on the right side (blue arrow), preserved buccal artery on the right side (green arrow), and absence of the facial vessels after iFAMM harvesting on the left side (red arrow)

**Fig. 6:**
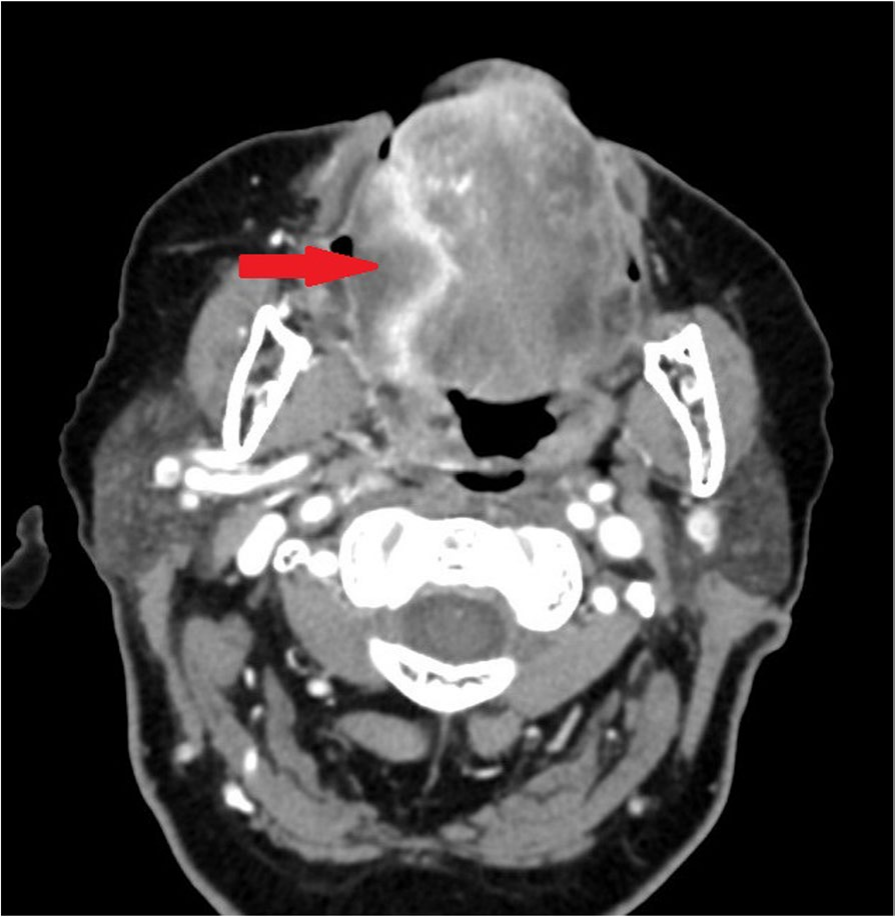
CT axial view of extensive local recurrence after radiotherapy of the tongue SCC on the right side (red arrow)

**Fig. 7:**
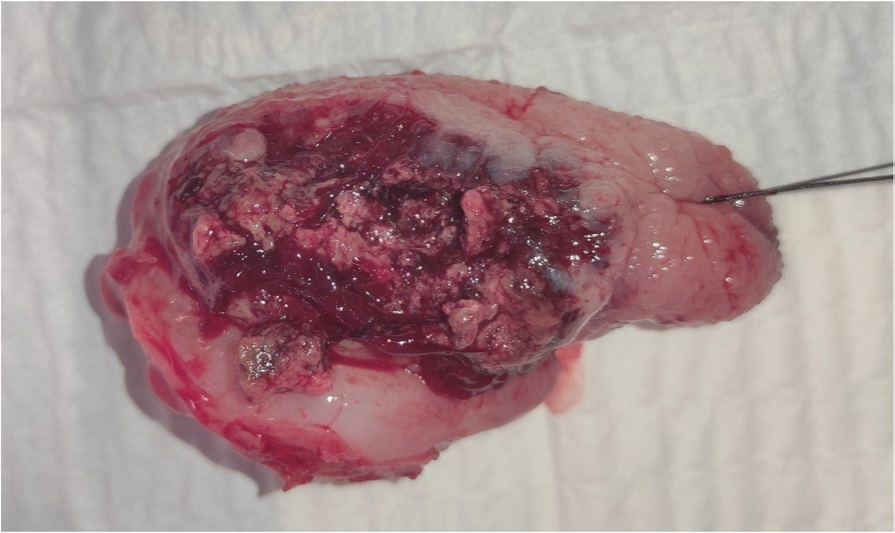
Salvage surgery. Surgical specimen of the tongue after right hemiglossectomy

**Fig. 8:**
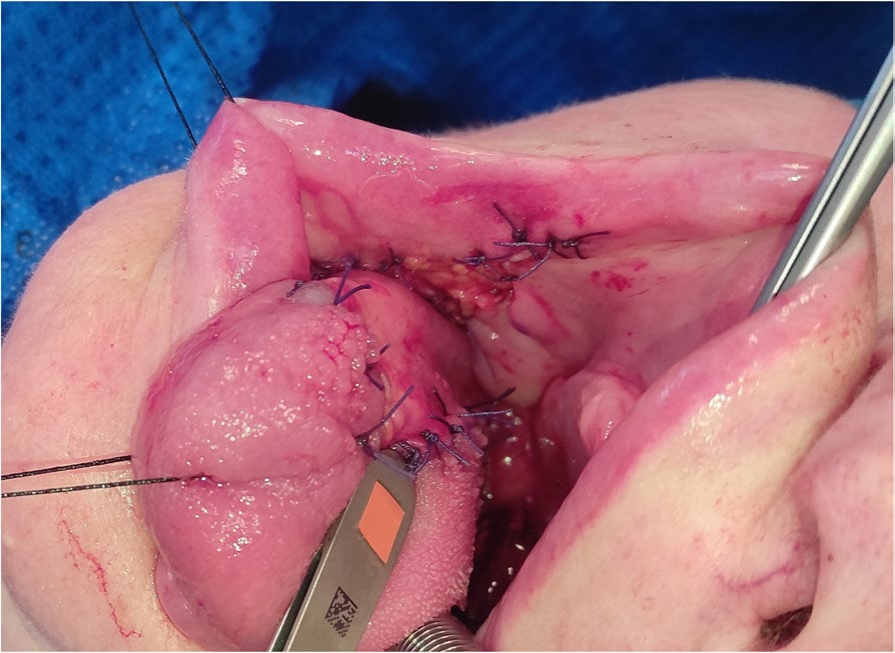
Immediate postoperative result of tongue reconstruction with dpFAMM flap

**Fig. 9:**
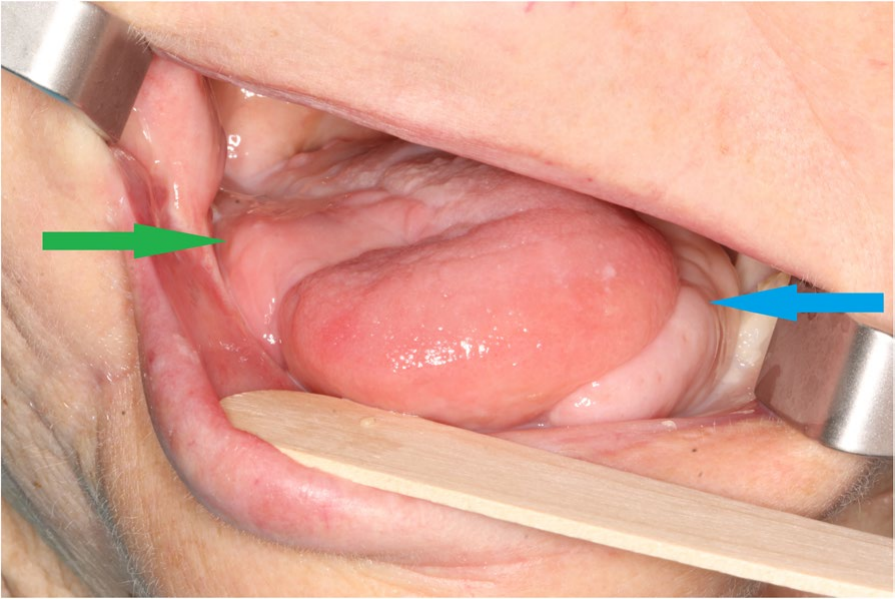
Final result. Three months after tongue reconstruction with dpFAMM flap (green arrow) and iFAMM flap (blue arrow—21 months after surgery)

In February 2020, the patient started hormone therapy with tamoxifen due to breast cancer and had oncological controls in our outpatient department every month. In December 2020, after 4 months from the last visit, the patient was admitted to the outpatient clinic with suspicion of tongue cancer on the opposite, right side. Biopsy from the ulceration confirmed SCC G2. Clinical examination and CT revealed only local advancement of the disease cT2N0M0. Breast cancer disease was stable. We proposed surgical treatment, but the patient again did not give her consent. For that reason, the patient was qualified for definitive radiotherapy. However, the total dose was reduced due to general health conditions. The patient received 30 Gy in 10 fractions on the second primary and cervical region, and the treatment was finished in February 2021. In May 2021, 2 months after the last visit, the patient was admitted to our outpatient department with dysphagia and odynophagia. Clinical examination and biopsy confirmed local recurrence of the tongue cancer on the right side. CT showed local recurrence with dimensions 21 × 42 × 29 mm and without suspicious neck lymph nodes (rT3N0M0) (Figs. 5 and 6). Definitive radiotherapy was impossible at this stage of the disease. The patients agreed to salvage surgery, hemiglossectomy with reconstruction and without neck dissection. Due to the fact that the right facial vessels were ligated in November 2019 and considering the extent of the defect, iFAMM was not possible. Also, the general condition of the patient was a contraindication to extensive, long procedures such as free flap reconstruction. Another contraindication for free flap reconstruction was a higher risk of early postoperative thrombosis of microvascular anastomoses due to continuous tamoxifen intake. Based on CT evaluation, we decided to use a dpFAMM flap for reconstruction, which was performed as described earlier (Figs. 7 and 8) [3]. Histopathological examination revealed SCC G2 resected with adequate margins >5mm (pT3). The healing process was uneventful (Fig. 9). Unfortunately, in August 2021, we observed enlarged cervical lymph nodes on the left side. Neck dissection in levels II and V was performed. Histopathological examination confirmed three metastatic lymph nodes. The patient was disqualified from adjuvant radiotherapy and chemotherapy. The patient died 2 months following neck dissection due to pneumonia.

## Discussion and conclusions

Soft tissue defects of the oral cavity are challenging in reconstructive surgery due to loss of motility, secretion, and sensory functions of the mucous membrane. Reconstruction especially of tongue defects should ensure proper patient’s speech and swallowing. Skin grafts are ineffective in the case of bone exposure and have a tendency to keratinization and cicatrization, which additionally decreases the movability of the remaining, healthy oral mucosa [8]. Also, most pedicled regional and free flaps containing skin islands are characterized by impaired sensitivity, keratinization, hair growth, and donor site morbidity. Local musculomucosal flaps are a good method of reconstruction of moderate tongue defects [3]. According to Massarelli et al. [8], all buccinator musculomucosal flaps provide proper mucus secretion and sensitivity without shrinking tendency, especially after radiotherapy.

The iFAMM flap in the tongue reconstruction is possible in case of facial vessel preservation. Massarelli et al. [8] suggest that iFAMM flap pedicled solely on the facial artery with the surrounding fat tissue provides the correct venous drainage, without flap congestion. However, Rahpeyma et al. [9] in experimental studies on dogs observed iFAMM flap loss in each case of facial vein ligation. For that reason, iFAMM flap, pedicled only on the facial artery, is not acceptable for clinical usage [9]. In our case, the patient had facial vein and artery ligated during neck dissection 18 months earlier. This was the main contraindication for tongue reconstruction by iFAMM. However, the vascularization of the buccinator muscle is derived from the branches of the facial vessel in the anterior part and buccal vessels in the posterior region. Due to the fact that CT revealed preserved distal part of the facial artery in the buccal region (Fig. 5) and a large number of anastomoses between facial and buccal angiosome, which ensures good blood supply through the buccal vessels, we decided to used dpFAMM flap for this reconstruction [8]. The dpFAMM flap combines the advantages of both the FAMM and the Bozola flap, which allows its extension to be increased with sufficient venous drainage [3].

Another issue concerning reconstruction is preoperative radiotherapy. It should be pointed out that both the donor and recipient sites comprise the irradiation field, which implies a higher risk of healing process problems with potential flap necrosis. O’Leary and Bundgaard [10] suggested that the FAMM flap is not suitable in patients, who underwent the previous radiotherapy, due to the risk of such complications as trismus, bleeding, osteoradionecrosis, and impaired healing followed by flap necrosis. They observed partial flap necrosis in 75% (3 from 4 patients) of cases. On the other hand, Ayad et al. [11] did not notice the specific complication rate in the group of 10 patients previously irradiated. In our case, we also did not detect problems with healing, bleeding, and proper mouth opening. The short term of the follow-up did not allow for evaluation of possible mandibular osteoradionecrosis.

The healing process can also be disturbed by hormone therapy. According to Billon et al. [12], hormone therapy, including tamoxifen intake, seems to be associated with a higher risk of postoperative wound healing complications in patients with breast reconstruction. In addition, Parikh et al. [13] in their meta-analysis concluded that perioperative tamoxifen therapy may increase the risk of thrombotic flap complications and flap loss in patients undergoing free flap reconstruction due to breast cancer. They suggested that short cessation of the tamoxifen therapy, about 4 weeks prior to reconstructive treatment, might decrease the risk of complications [13]. Our patient also suffered from synchronous mucinous carcinoma of the left breast treated with tamoxifen 15 months before salvage surgery. This was one of the contraindications for free radial forearm flap application and choice of dpFAMM flap for tongue reconstruction. Transient discontinuation of tamoxifen before surgery was not recommended. However, the healing process was uneventful.

The extension of the dpFAMM flap in the anterior part of the buccinator muscle provided an ability for tongue reconstruction, even after hemiglossectomy. If the apex of the tongue can be preserved during ablative surgery, like in this case, the dpFAMM flap can be doubled. The preserved apex of the tongue is rotated backward to obtain the best clinical result of the reconstruction. Flap harvesting takes around 30–50 min and can be done by one surgical team. The dpFAMM flap has a perfect color and structure, matching the surrounding tissues without additional extraoral scars. This case presentation only highlights that the dpFAMM flap can be used for reconstruction in salvage surgery, even, if the facial vessels had been ligated previously and the patient was irradiated and treated with hormone therapy. However, further studies with more patients must be conducted to prove the utility of the dpFAMM flap in such cases. Other advantages of the dpFAMM flap include its feasibility in the reconstruction of other areas, like the floor of the mouth, the alveolar ridge, the soft and hard palates, or the oropharynx.

## Data Availability

All data during the study are included within the article.

## Abbreviations

FAMM: Facial artery musculomucosal flap
dpFAMM: Double-pedicled facial artery musculomucosal flap
iFAMM: Island facial artery musculomucosal flap
SCC: Squamous cell carcinoma
CT: Computer tomography
